# Regulation and overexpression studies of YidC in *Mycobacterium tuberculosis*

**DOI:** 10.1038/s41598-018-35475-4

**Published:** 2018-11-20

**Authors:** Preeti Thakur, Eira Choudhary, Madhu Pareek, Nisheeth Agarwal

**Affiliations:** Translational Health Science and Technology Institute, NCR Biotech Science Cluster, 3rd Milestone, Faridabad–Gurgaon Expressway, Faridabad, 121001 Haryana India

## Abstract

The preprotein translocase, YidC is an envelope protein which controls respiratory metabolism in *Mycobacterium tuberculosis*. Previously, we have established that depletion of *yidC* is deleterious for both extra- and intracellular proliferation of *M*. *tuberculosis*; however, it remains unclear how YidC expression is regulated under different growth conditions and whether its altered expression impact mycobacterial physiology. Herein, we show that *yidC* is expressed as an operon with upstream genes. Interestingly, expression analysis under various stress conditions reveals a distinct paradox in the profile of the *yidC* mRNA transcripts and the YidC protein. While YidC protein level is moderately elevated upon bacterial exposure to cell surface stresses, the corresponding mRNA transcript levels are significantly repressed under these conditions. In contrast, overexpression of *M*. *tuberculosis yidC* under a strong anhydrotetracycline-inducible promoter results in significant induction of YidC protein. Additionally, we also observe that overexpression of *M*. *tuberculosis yidC*, and not of its counterpart from fast-growing *M*. *smegmatis*, results in altered *in vitro* growth of bacteria, compromised integrity of bacterial cell envelope and differential expression of a small set of genes including those which are regulated under detergent stress. Overall findings of our study suggest that YidC proteins of slow- and fast-growing mycobacteria are functionally distinct despite exhibiting a great deal of identity.

## Introduction

Tuberculosis (TB), which is caused by pathogen *Mycobacterium tuberculosis*, is one of the leading causes of human death by an infectious agent in the world. According to a recent estimate by the World Health Organization (WHO), 10.4 million people suffered from TB and 1.8 million died from the disease in 2015^1^. In the immunocompetent individuals, *M*. *tuberculosis* infection is primarily controlled by alveolar macrophages which engulf pathogen by surface exposed, vesicular, or cytoplasmic pattern recognition receptors specifically recognizing microbe-associated molecular patterns on bacteria^2^. Subsequently, macrophages exhibit a network of activities such as generation of free radicals, a hostile acidic environment, quenching of essential nutrients, production of anti-mycobacterial peptides and suicidal activities such as autophagy, necroptosis, or apoptosis to prevent spread of infection^3^. Another factor that is important in moderating the interaction of *Mycobacterium* with alveolar macrophages is pulmonary surfactant, a complex mixture of lipids and proteins secreted by type II epithelial cells in the alveolar lining, which bacteria frequently encounter and interact with^4–7^. Exposure to surfactant causes cell surface stress in *M*. *tuberculosis* which subsequently influences the mycobacterial physiology and its interaction with macrophages^8^. Despite these rigorous responses by the host macrophages, *M*. *tuberculosis* exhibits various counter-strategies by quickly modulating the expression of key virulence genes to replicate and persist intracellularly^9^. Hence, there are ongoing interactions between *M*. *tuberculosis* and pulmonary macrophages which play a crucial role in deciding the fate of disease caused by this microorganism.

Export of proteins from the cytoplasm to the cell surface of bacteria or their secretion into the host is critical for host-pathogen interaction and long-term intracellular survival of bacteria^10,11^. In *M*. *tuberculosis*, the majority of proteins are exported to envelope primarily by the Sec and the Tat translocation pathways, whereas a set of secretory proteins rely on specialized machineries such as the SecA2 and ESX, also referred as Type VII, secretion systems for their secretion in the extracellular environment. Importantly, several of the exported proteins are essential for *M*. *tuberculosis* virulence and intracellular survival^11–13^. Recently, we have characterized the role of another cell envelope-localized translocase, YidC in export of essential subunits of respiratory chain proteins such as succinate dehydrogenase subunits A_1 and B_1 and ubiquinol-cytochrome C reductase subunit QcrA to cell envelope of *M*. *tuberculosis*. We observed that depletion of YidC modulates the expression of genes involved in respiratory metabolism which subsequently causes redox imbalance and affects bacterial survival *in vitro* and in macrophages^14^. While, these observations are reminiscent of a crucial role of YidC translocase in the *in vitro* and intracellular survival of *M*. *tuberculosis*, we are far from understanding the importance of this envelope protein in mycobacteria during stress.

Here, we study the expression profile of YidC under different stress conditions and the effect of altered YidC levels on mycobacterial physiology. Since *yidC* locus is 100% conserved in virulent and avirulent strains of *M*. *tuberculosis*, we restricted our study to avirulent *M*. *tuberculosis* H37Ra. Expression analysis of mRNA transcripts indicated that *yidC* is transcribed as an operon with upstream genes *MRA_3961* (orthologue of *Rv3922c* in *M*. *tuberculosis* H37Rv), *rnpA* and *rpmH*. Remarkably, bacterial treatment with various stress agents reveals a contrasting effect on the expression of the *yidC* mRNA transcripts and the corresponding protein. Although YidC protein levels are moderately induced upon exposure to cell surface stress agents, the same stress conditions resulted in significant drop in its transcript levels. In contrast, overexpression of *yidC* transcripts under the anhydrotetracycline (ATc)-inducible promoter using a replicative plasmid leads to significant induction of the YidC protein in both *M*. *tuberculosis* as well as in fast-growing *M*. *smegmatis* bacteria. Interestingly, we observed that controlled expression of *M*. *tuberculosis* YidC is essential for optimal growth of bacteria as its induction stalls bacterial replication and elicits membrane stress as evidenced by disruption of the cell envelope and induction of a set of cell-surface stress regulons. Importantly, these features are restricted to only *M*. *tuberculosis* YidC protein as similar expression level of its counterpart from *M*. *smegmatis* fails to show any effect on bacterial growth and metabolism.

## Results

### *M*. *tuberculosis yidC* is transcribed as an operon

**Figure 1:**
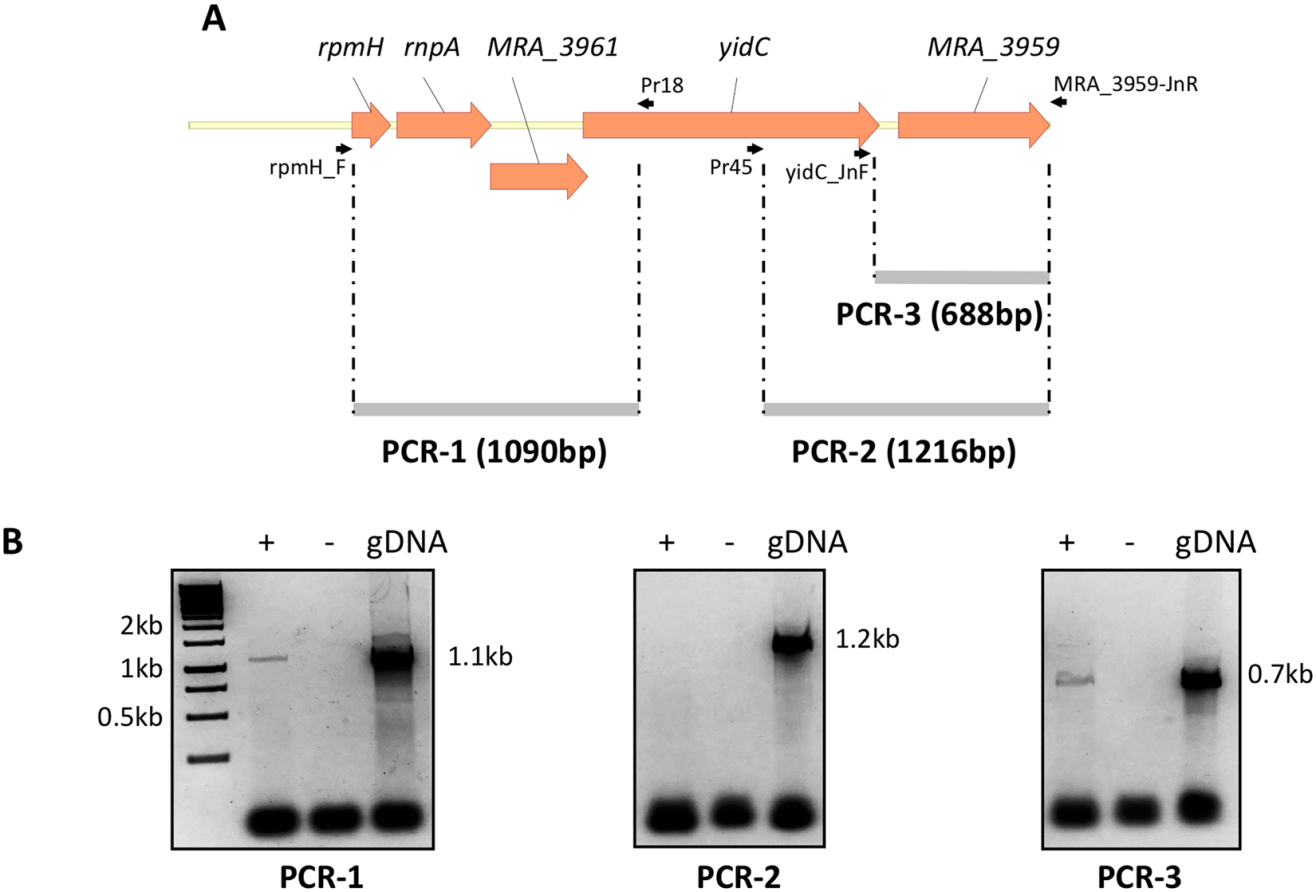
Analysis of the *yidC* mRNA transcripts in *M*. *tuberculosis*. Transcript of *yidC*, prepared from wild-type *M*. *tuberculosis*, was subjected to reverse transcription for the cDNA synthesis followed by PCR amplification of the junction sequences across various ORFs in the locus using designated primer pairs, as indicated (**A**). Presence of amplicon of ~1.1 kb in the reaction PCR-1 in the presence of reverse transcriptase (RT, +) suggests expression of *yidC* as an operon with upstream genes, whereas no amplification was observed in the reaction PCR-2 indicating that the downstream gene *MRA_3959* is transcribed from an independent promoter situated upstream to its ORF; independent expression of *MRA_3959* is also verified by PCR-3 indicating the amplification of expected size of ~700 bp (**B**). Reactions performed without RT (-) in (**B**) were used as negative control to verify specificity of RT-PCR, whereas amplification with genomic DNA (gDNA) was used as positive control for each primer pair.

*In silico* analysis of *yidC* locus indicates the presence of 3 open reading frames (ORFs) upstream to *yidC* namely, *MRA_3961*, *rnpA* and *rpmH*; while sequences of *rnpA*, *MRA_3961* and *yidC* ORFs overlap each other, the first ORF *rpmH* in the locus is only 23 bp apart from the downstream gene (Fig. 1A). To determine if any or all of these genes are transcribed in an operon, we amplified the entire locus using the cDNA as template, and *rpmH*-specific forward primer rpmH_F and *yidC*-specific reverse primer, Pr18 (Fig. 1A and Table 1). Presence of a 1090 bp long amplicon exclusively in the presence of reverse transcriptase (RT+) or with genomic DNA (gDNA), but not in its absence (RT−) suggests that these genes are indeed transcribed as an operon from a common promoter situated upstream to the first gene, *rpmH* (PCR–1, Fig. 1B). In contrast, we could not observe amplification of expected size of ~1.2 kb with cDNA using primer pairs Pr45–MRA_3959–JnR binding to the sequence 546 bp downstream to the 5′ end of *yidC* and extreme 3′ region of *MRA_3959*, respectively (PCR–2, RT+ Fig. 1B), which otherwise yielded the PCR product of anticipated length with gDNA (PCR–2, gDNA Fig. 1B), thus indicating that transcription of downstream gene *MRA_3959* is independent of *yidC*. Presence of amplicon of ~700 bp with forward primer, yidC_JnF (which hybridizes just 54 bp upstream to the 3′ end of *yidC*) and reverse primer MRA_3959–JnR in the reaction mix containing RT enzyme (RT+) (PCR-3, Fig. 1B) further reiterates that *MRA_3959* does not belong to *yidC* operon, and is possibly transcribed as an independent unit by using a promoter which overlaps with the 3′ end of the *yidC* ORF.

**Table 1:** List of plasmids and primers.

A. Primers			
S. No.	Primer Name	Sequence (5′-3′)	Reference
1.	Pr17	GTTTGATTTCTTCAGTCTCG	^14^(Forward primer for qRT-PCR analysis of *yidC*(*Mtb*))
2.	Pr18	TTGATCTGTGGTTGCAGTTC	^14^(Reverse primer for qRT-PCR analysis of *yidC*(*Mtb*))
3.	Pr3	GATCCATATGAGTCTTTTGTTTGATTTC	^14^(Forward primer for PCR amplification of *yidC*(*Mtb*) ORF)
4.	Pr5	GCGCAAGCTTTCAACGTTTGCGTTTTTTCGGTC	^17^(Reverse primer for PCR amplification of *yidC*(*Mtb*) ORF)
5.	rpmH-F	GATCCATATGACCAAGGGCAAAAGGACCTTCCAG	This study
6.	Pr45	ATGACGCAACGGTCCGGGTTG	This study
7.	MRA_3959-JnR	CTAGTCGCGGAGCACAACG	This study
8.	yidC-JnF	CCGAGCGCCCGTACGCCGCG	This study
9.	MS6942_F	GCGCCATATGTTTAACTTCTTCAGCTTG	This study (For PCR amplification of *yidC*(*Msm*) ORF)
10.	MS6942_R	GCGCAAGCTTTCATCGTTTCCGCTTCTTGG	
11.	MS6942_RTF	AAGCTCGCGCTCTACGTGTTCC	This study (For qRT-PCR analysis of *yidC*(*Msm*))
12.	MS6942_RTR	TCATCCGATTCGGCTTGGCG	
13.	Rv0251c_RTF	CAATCTCGCATTGTGGTCGC	This study (For qRT-PCR analysis of *Rv0251c*)
14.	Rv0251c_RTR	TTGTCGACGTCAATGCCGGG	
15.	Rv0341c_RTF	AGTGCCGGGCTGATCGATATCG	This study (For qRT-PCR analysis of *Rv0341c*)
16.	Rv0341c_RTR	TCGTGATGACGCTTGCCACC	
17.	Rv0676c_RTF	ATGATCGTGCAAAGGACAGC	This study (For qRT-PCR analysis of *Rv0676c*)
18.	Rv0676c_RTR	ACACCGCCTGTATCTGTCCG	
19.	Rv1221_RTF	CGGGTTGGGAATACGGAATCG	This study (For qRT-PCR analysis of *Rv1221*)
20.	Rv1221_RTR	CCTGCAATTGGTCAGACGGC	
21.	Rv1909_RTF	CCTCTATACCGGACTACGCC	This study (For qRT-PCR analysis of *Rv1909*)
22.	Rv1909_RTR	AGGGTTGGATCTTTCGCACC	
23.	Rv2590_RTF	TCGAGGACCTATACGCCAGC	This study (For qRT-PCR analysis of *Rv2590*)
24.	Rv2590_RTR	AGTAGCTCGACCATGGTGCG	
25.	Rv2710_RTF	GGGTTGACAGCGATCTGGATGC	This study (For qRT-PCR analysis of *sigB*)
26.	Rv2710_RTR	GGTCGCGTTTTCGGTTCTCG	
27.	Rv2744c_RTF	ATGGCCAATCCGTTCGTTAAAGC	This study (For qRT-PCR analysis of *Rv2744c*)
28.	Rv2744c_RTR	GCATCTCCAATTGACGCTGG	
29.	Rv3862c_RTF	CATGACAGTAACCGCCCTGT	This study (For qRT-PCR analysis of *whiB6*)
30.	Rv3862c_RTR	TTCGGGAATTACGACCCCTG	
**B. Plasmids**			
**S. No**.	**Name**	**Plasmid description**	**Reference/Source**
1.	pGEM-T^®^ Easy	Plasmid with *E*. *coli* Ori for TA Cloning	Promega
2.	pGEM-*yidC*(*Msm*)	Derivative of pGEM- T^®^ Easy harbouring MSMEG_6942 ORF	This study
3.	pGEM-*yidC*(*Mtb*)	Derivative of pGEM- T^®^ Easy harbouring *MRA_3960* ORF	This study
4.	pTetR	Replicative Mycobacteria- *E*. *coli* shuttle plasmid for ATc inducible expression of genes in mycobacteria	^17^
5.	pTetR-*yidC*(*Msm*)	Derivative of pTetR harbouring *MSMEG_6942* ORF at *Nde I-Hind III* downstream to ATc-inducible promoter	This study
6.	pTetR-*yidC*(*Mtb*)	Derivative of pTetR harbouring *MRA_3960* ORF at *Nde I-Hind III* downstream to ATc-inducible promoter	This study

### Effect of stress exposure on YidC protein expression

**Figure 2:**
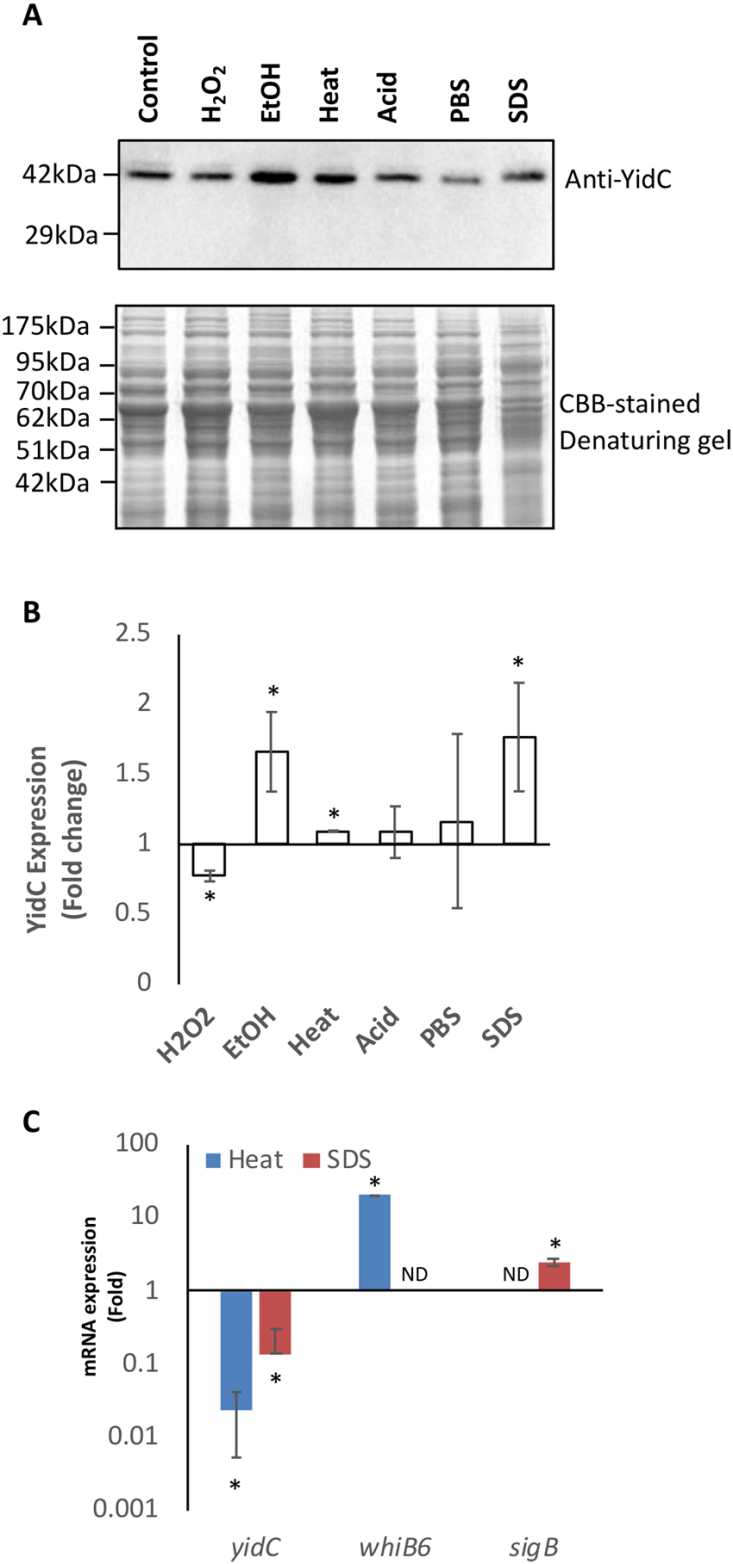
Analysis of YidC expression under stress. To monitor YidC production under stress, bacterial cultures at OD_600_ of ~0.8 were exposed to different stress agents for 24 hours followed by immunoblotting of WCEs with anti-YidC antibodies. Differences in YidC levels between treated and untreated cultures were calculated after normalizing the band intensities of the anti-YidC blot with total protein in the corresponding lysates, as observed in the CBB-stained gel shown below the immunoblot (**A**). Band intensities were determined by densitometric scanning using the NIH ImageJ tool (**B**). Effect of cell surface stresses such as detergent (0.1% SDS) and heat (45 °C) on mRNA transcript of *yidC* was examined by qRT-PCR (**C**). Fold change in expression levels under each of these conditions was calculated after normalization with the levels of *sigA* in the same samples. Primers specific to *whiB6* and *sigB* (Table 1) were used as positive control for heat and detergent stress, respectively. ND: not-determined. Blots represent three repeat experiments in (**A**). Bar graphs in (**B**) and (**C**) were obtained by plotting mean ± s.d. of three measurements. Asterisks indicate *P* ≤ 0.05, as determined by Student’s *t* test.

Previously we have shown that envelope-localized YidC translocase is required for both extra- and intracellular growth of *M*. *tuberculosis*^14^. While YidC is critical for maintaining respiratory metabolism during the extracellular growth of bacteria, it is not clear how this protein protects *Mycobacterium* from the host assaults during infection. In order to gain an insight into the requirement of YidC in host-*M*. *tuberculosis* interaction, we examined status of the YidC protein level under various stress conditions that mimic the intracellular environment faced by the pathogen. These include phosphate buffered saline (PBS, nutritional starvation), H_2_O_2_ (oxidative stress), pH4.5 (acidic stress), heat (45 °C), 0.1% SDS and 5% ethanol (cell surface stress). Bacterial culture was grown in 7H9-OADS medium to OD_600_ of 0.8 and divided into multiple aliquots, each exposed to these stress agents for 24 hours, followed by immunoblotting of whole cell extracts (WCEs) with anti-YidC antibodies. Fold-changes in YidC levels under different stress conditions relative to its expression in the untreated cells was measured by densitometric scanning of the immunoblot. Values were obtained after normalization with the band intensities of the corresponding lysates in Coomassie Brilliant Blue (CBB)-stained gel. As depicted in Fig. 2, expression of YidC is increased by 70-, 10- and 80% respectively after exposure to cell surface stress agents ethanol, heat and SDS. Conversely, YidC expression is mildly reduced (by 23%) in H_2_O_2_-treated mycobacteria whereas no significant change is observed following acid- or PBS treatment (Fig. 2A-B).

### Expression of *yidC* mRNA transcripts is adversely affected under stress

In order to test if elevated levels of YidC under cell surface stress is due to its regulation at the transcript level, we next examined the expression of *yidC* mRNA transcripts under these conditions by quantitative reverse transcription real-time PCR (qRT-PCR). Surprisingly, in contrast to YidC protein, levels of the corresponding mRNA transcripts are significantly repressed in *M*. *tuberculosis* upon 24 hours of exposure to heat (45 °C) and SDS (0.1%). As shown in Fig. 2C, exposure of bacteria to high temperature causes ~45-fold reduction in *yidC* mRNA transcripts, whereas the same results in induction of an unrelated gene *whiB6* by ~10-fold, as reported previously^15^. A similar effect was also observed upon SDS treatment which caused ~10-fold reduction in *yidC* transcript levels as against 5-fold induction of control *sigB* transcripts^16^ (Fig. 2C).

The paradoxical effects of stresses on the *yidC* transcripts and the YidC protein further suggest that the expression of YidC preprotein translocase is regulated at multiple levels of transcription and translation in *M*. *tuberculosis*.

### Consequence of overexpression of the *yidC* mRNA transcripts on the YidC protein translation in mycobacteria

Intrigued with the above results, we next examined if the apparent paradox in the expression pattern of *yidC* transcripts and the YidC protein stems from its locus on the genome or is an inherent feature of the *yidC* ORF. To address this, we overexpressed *yidC* of *M*. *tuberculosis* (annotated hereafter as *yidC(Mtb)*) under a strong ATc-regulated promoter *P*_*myc1TetO*_ using the replicative plasmid pTetR. An orthologue of *yidC(Mtb)* in the fast-growing bacterium *M*. *smegmatis*, *MSMEG_6942* (termed as *yidC(Msm)*) which shares 75% identity with *yidC(Mtb)*, was simultaneously used as control. Full-length genes *yidC(Mtb)* and *yidC(Msm)* were cloned in the *Escherichia coli*-mycobacteria shuttle plasmid pTetR (Table 1) downstream to *P*_*myc1TetO*_^17^ and the recombinant pTetR-*yidC(Msm)/ yidC(Mtb)* and empty pTetR plasmids were then transformed in *M*. *smegmatis* MC^2^155 and *M*. *tuberculosis* H37Ra, respectively.

**Figure 3:**
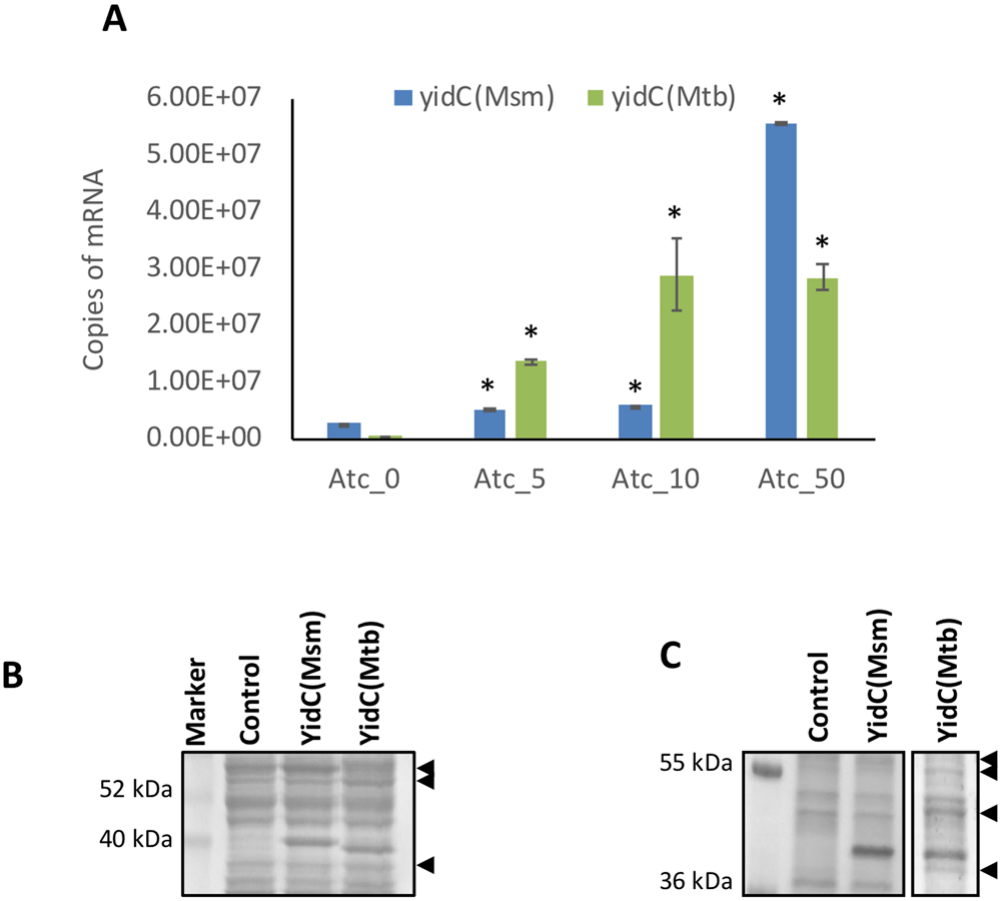
Overexpression of the *yidC* mRNA transcripts and subsequent effect on the YidC protein synthesis in mycobacteria. Cultures of *M*. *smegmatis* harbouring pTetR::*yidC(Msm)* or pTetR::*yidC(Mtb)* were treated with different concentrations of ATc (0–50 ng/ml) for 24 hours and copy number of respective mRNA transcripts was measured by qRT-PCR using standards prepared with known concentrations of gnomic DNA, as described earlier^17^ (**A**). Effect of overexpression of mRNA transcripts of both *yidC(Msm)* and *yidC(Mtb)* on the levels of the respective YidC proteins was examined, in *M*. *smegmatis* (**B**) or in *M*. *tuberculosis* (**C**). Shown are the envelope proteins of bacteria (20 µg/lane) in CBB-stained denaturing polyacrylamide gels. Mean ± s.d. of three independent experiments is shown in (**A**). Asterisks represent level of significance as determined by paired Student’s t-test (*P* < 0.05). Arrowheads mark the envelope proteins that are altered upon YidC(Mtb) induction. Images in (**B-C**) represent 3 biological replicates.

In order to test the effect of induction of *yidC(Mtb)* and *yidC(Msm)* on synthesis of the respective proteins, firstly we estimated the copy number of respective mRNA transcripts in *M*. *smegmatis* after 24 hours of treatment with different doses of ATc. As can be seen in Fig. 3A, there is a gradual increase in transcript levels of both the genes with increasing ATc. With respect to ATc-untreated bacteria, level of *yidC(Msm)* is increased in *M*. *smegmatis*::pTetR-*yidC(Msm)* by 1.9, 2.2 and 20.7-fold after treatment with 5, 10 and 50 ng/ml ATc, respectively. On the other hand, the heterologous gene *yidC(Mtb)* exhibits 99-, 211 and 209 fold induction in *M*. *smegmatis*::pTetR-*yidC(Mtb)* after treatment with 5, 10 and 50 ng/ml ATc, respectively. Importantly, despite significant induction of *yidC(Mtb)*, number of total (genomic and plasmid-borne) *yidC(Msm)* transcripts remains ~2-fold higher than of *yidC(Mtb)* at 50 ng/ml ATc-treatment (Fig. 3A). Moreover, we did not see any effect of ATc on the level of *yidC* in cells harbouring empty plasmid pTetR.

Once we achieved the induction of mRNA transcripts, we subsequently analysed the expression of the respective YidC proteins by CBB staining of the denaturing polyacrylamide gels. Total cell lysates prepared from *M*. *smegmatis*::pTetR, *M*. *smegmatis*::pTetR-*yidC(Msm)*, and *M*. *smegmatis*::pTetR-*yidC(Mtb)* after 50 ng/ml ATc treatment for 24 hours were fractionated to extract the cell envelope proteins followed by equal loading of samples. Interestingly, the polyacrylamide gel shows similar level of induction and localization of the YidC(Msm) and YidC(Mtb) migrating at ~40 kDa in the respective strains of *M*. *smegmatis*, whereas no band was seen at the similar position in the pTetR-containing control bacteria (Fig. 3B). Equal induction of the two proteins in *M*. *smegmatis* was also confirmed by anti-YidC immunoblotting of the respective lysates (Supplementary Fig. 1A). Importantly, we find a minor difference in the migration of YidC(Msm) and YidC(Mtb) on the denaturing gel, which is not due to their overexpression as similar level of change in mobility is also observed with the native proteins by anti-YidC immunoblotting of WCEs from the wild-type bacteria (Supplementary Fig. 1B). The anomalous migration on SDS-PAGE of the two membrane proteins from different mycobacterial species is most likely due to their altered amino acid sequences (Supplementary Fig. 2A). Akin to these results, we also observe a significant induction of both the proteins in *M*. *tuberculosis* transformed with pTetR-*yidC(Msm)* and pTetR-*yidC(Mtb)* after 4 days of treatment with 50 ng/ml ATc (Fig. 3C), which lasts for up to one week. Overall, the above findings clearly indicate that plasmid-mediated induction of *yidC* mRNA transcripts results in matching levels of production of YidC proteins in both the fast- and slow-growing mycobacteria.

### Effect of YidC overexpression on *in vitro* growth of mycobacteria

**Figure 4:**
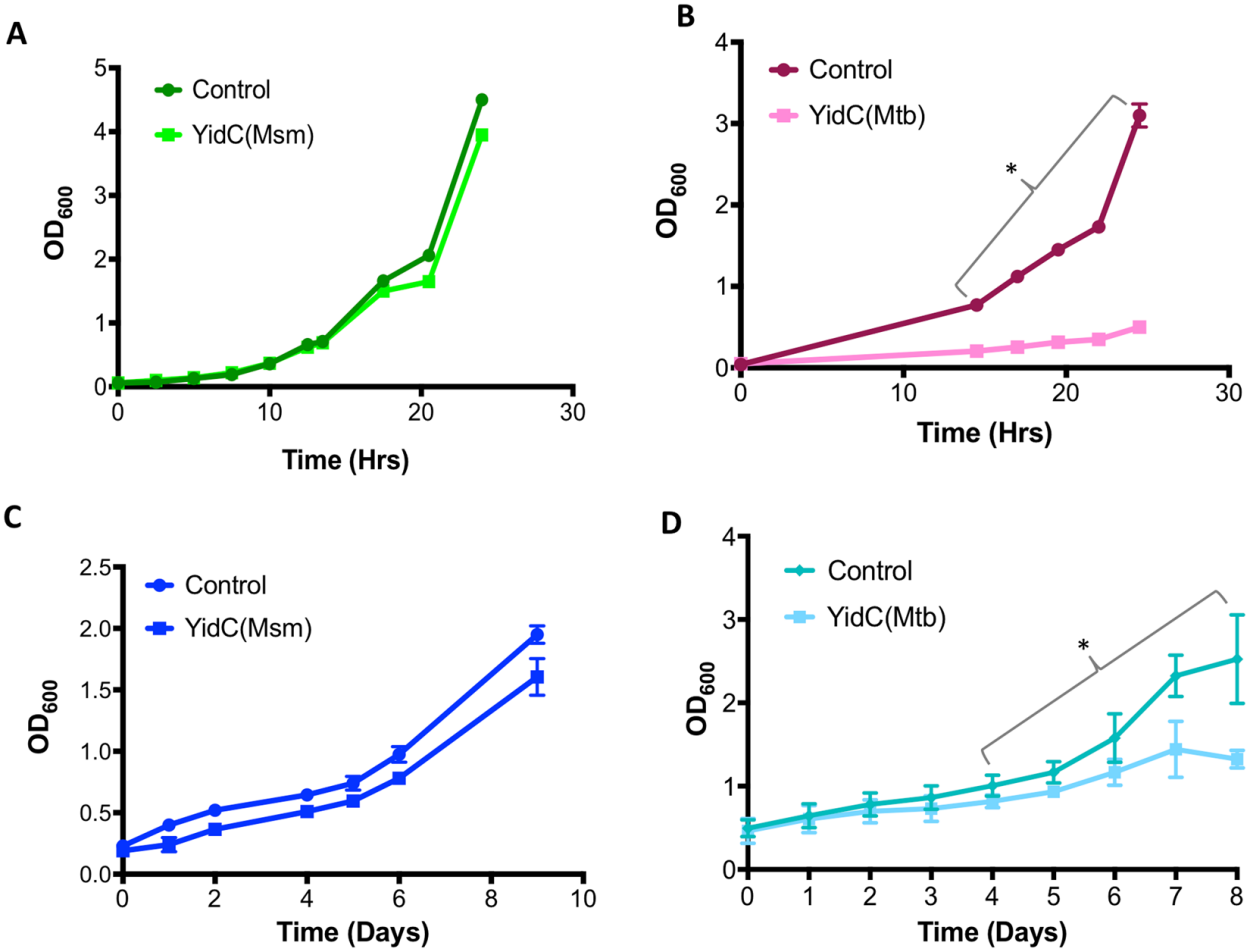
Effect of overexpression of YidC on *in vitro* growth of bacteria. Bacterial growth was observed by measuring OD_600_ of broth cultures of *M*. *smegmatis* (**A**,**B**) or *M*. *tuberculosis* (**C**,**D**) transformed with either empty vector (control), pTetR-*yidC(Msm)* or pTetR-*yidC(Mtb)* treated with 50 ng/ml ATc for designated time points. Mean values ± s.d. of at least three repeat experiments are shown. Asterisks represent level of significance as determined by paired Student’s t-test (*P* < 0.05).

While analysing the expression of YidC, we made a striking observation which reveals distinct effect of YidC(Mtb) induction on proteome profile of cell envelope (Fig. 3B-C) and *in vitro* growth of mycobacteria. To gain further insights into the effect of YidC induction on mycobacterial physiology, we systematically analysed growth of bacteria overexpressing YidC. Cultures of *M*. *smegmatis* transformed with pTetR (control), pTetR-*yidC(Msm)* (YidC(Msm)) or pTetR-*yidC(Mtb)* (YidC(Mtb)) in 7H9 broth were treated with 50 ng/ml ATc, and growth was monitored at regular time intervals by measuring OD_600_. Interestingly, it was observed that bacterial growth is severely inhibited upon induction of YidC(Mtb), whereas no major effect was noticed on growth of YidC(Msm)-expressing bacteria that exhibit growth profile similar to control strain (Fig. 4A-B). To further investigate if the effects of YidC overexpression on bacterial proliferation is universal to other species, we determined growth of *M*. *tuberculosis* overexpressing YidC(Msm) and YidC(Mtb), respectively. *M*. *tuberculosis* strains harbouring pTetR (control), pTetR-*yidC(Msm)* or pTetR-*yidC(Mtb)* were cultured in 7H9 broth and growth was examined in the presence of ATc by measuring OD_600_ of the bacterial cultures at regular time intervals. Bacterial cultures were first grown to OD_600_ of ~0.2–0.4 followed by addition of 50 ng/ml ATc. As shown in Fig. 4C, YidC(Msm)-induced culture of *M*. *tuberculosis* exhibits similar growth rate as observed with the control. In contrast, induction of YidC(Mtb) results in gradual reduction in growth which is evident as early as 2 days post-ATc treatment, whereas there is no change in growth profile of control bacteria. These differences become more significant at later time points from day 4 onwards (*P* ≤ 0.05) (Fig. 4D). Together, the above results suggest that any change in YidC(Mtb) expression beyond its native level causes an adverse effect on bacterial proliferation, as against YidC(Msm) which does not modulate mycobacterial replication.

### Overexpression of YidC(Mtb) modulates the expression of detergent-regulated genes

**Figure 5:**
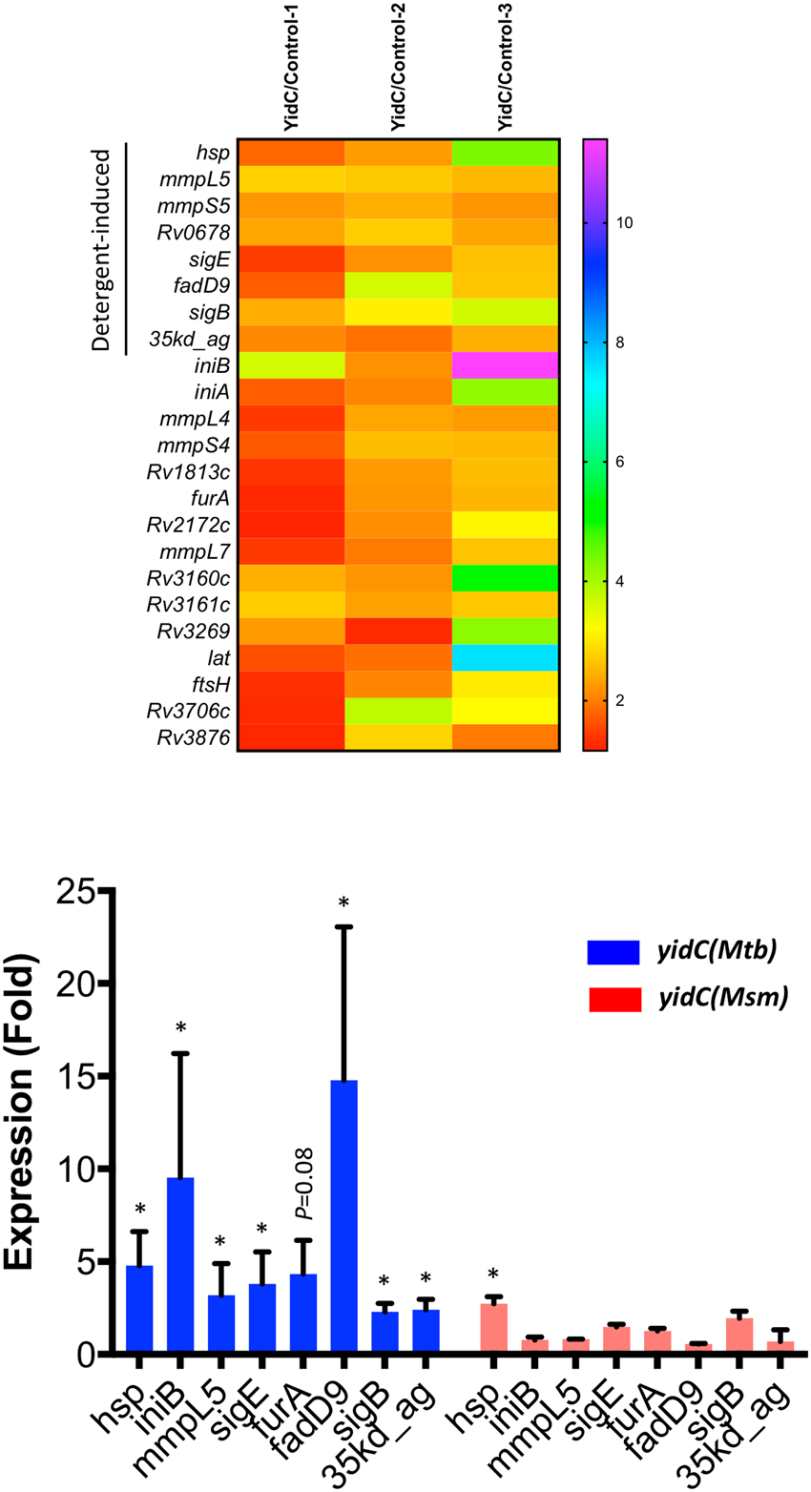
Influence of YidC-overexpression on global gene expression profile of *M*. *tuberculosis*. Heat map shows the status of differentially expressed genes in the microarray analysis of *M*. *tuberculosis*::pTetR-*yidC(Mtb)* compared to control after 4 days of treatment with 50 ng/ml ATc (**A**). Whole-genome transcriptional analysis was performed by microarray hybridization using arrays specific to *M*. *tuberculosis* H37Rv (Microarray Inc., USA). Increasing expression levels are denoted by different colours ranging from red to pink, respectively. Genes that exhibit >2-fold difference in expression between the two strains consistently in three biological replicates with *P* values of ≤ 0.05 and FDR of ≤ 5% are included in (**A**). As shown, overexpression of *yidC(Mtb)* results in induction of cell surface stress regulons, known to respond to cell surface stresses like SDS, indicating overexpression of *yidC(Mtb)* affects cell envelope integrity of *M*. *tuberculosis*. Differential expression of select genes including those regulated by SDS-treatment^16^, was also determined by qRT-PCR using RNA samples prepared from *M*. *tuberculosis* expressing *yidC(Mtb)* or *yidC(Msm)* and expression values in *yidC*-overexpressing cells relative to control are plotted for a comparative analysis (**B**). Mean ± s.d. of three independent experiments is shown. Asterisks represent level of significance as determined by paired Student’s t-test (*P* < 0.05).

Growth defect in *M*. *tuberculosis* because of YidC(Mtb) overexpression indicates perturbation of critical metabolic activity. To further understand the physiological basis of this phenotype, we analysed the whole-genome transcriptional profile of *M*. *tuberculosis* overexpressing *yidC(Mtb)*. Total RNA was extracted from log-phase cultures of control and *yidC(Mtb)*-overexpression strains after 4 days of treatment with 50 ng/ml ATc, and cDNA was synthesized using nucleotides’ mixture containing dCTP- tagged with Alexa Fluor 555 or 647 dyes, respectively. Equal amount of labelled cDNAs prepared from the two strains were mixed and subjected to microarray hybridization, as described in the materials and methods. Fluorescence intensities of each of the 3924 genes representing complete genome of *M*. *tuberculosis* in triplicates were analysed after normalization with total fluorescence intensities from three independent experiments, and fold-change in expression levels was determined. Figure 5A lists 23 genes that were differentially expressed by ≥2-fold upon *yidC(Mtb)* overexpression, consistently in three different experiments. Some of these genes were also validated by qRT-PCR using the gene-specific primers (Fig. 5B). Moreover, 33% of these genes which include *hsp*, *mmpL5*, *mmpS5*, *Rv0678*, *sigE*, *fadD9*, *sigB* and *pspA* are also reportedly induced by detergent stress, which destroys membrane integrity of mycobacteria^16^. Importantly, the effect on the expression of these genes is very specific to YidC(Mtb), since similar induction of YidC(Msm) in *M*. *tuberculosis* does not bring any major change in their levels (Fig. 5B).

### Modulation of YidC(Mtb) levels alters integrity of mycobacterial cell envelope

**Figure 6:**
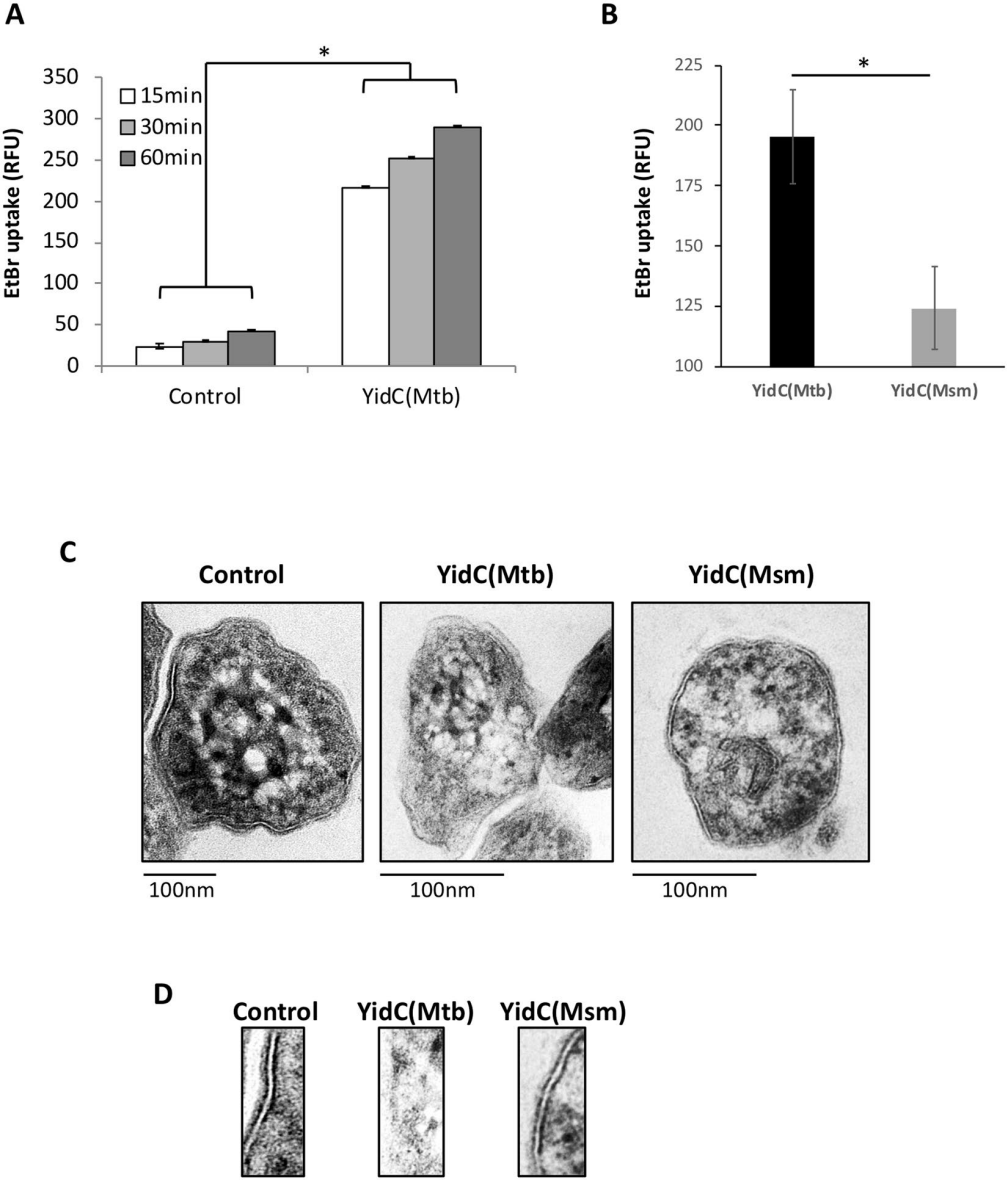
Modulation in YidC expression perturbs integrity of mycobacterial cell envelope. Relatively higher uptake of EtBr (**A,B**) and ruptured lipid bilayer (**C,D**) of bacteria overexpressing full length YidC(Mtb) compared to empty plasmid control or cells expressing YidC(Msm) indicate that YidC(Mtb) induction causes a membrane stress on bacteria. Induction of YidC was performed for 4 days in *M*. *tuberculosis* and for 24 hours in *M*. *smegmatis*. EtBr uptake was measured either for 15–60 mins in *M*. *tuberculosis* (**A**) or for 15 mins in *M*. *smegmatis* (**B**) cells overexpressing YidC, whereas cellular morphology was performed by transmission electron microscopy of *M*. *tuberculosis* (**C**). An enlarged image depicting portion of cell envelope of different strains is shown in (**D**). Mean ± s.d. of three independent experiments is shown in (**A,B**), whereas images in (**C**) represent 3 different fields, each from 2 biological replicates. Asterisks represent level of significance as determined by paired Student’s t-test (*P* < 0.05).

Transcriptional profiling by microarray highlights that overexpression of YidC is sensed as a membrane stress in *M*. *tuberculosis*, which may have resulted in weakening of cell envelope. In order to substantiate this assumption, we measured uptake of ethidium bromide (EtBr) across the mycobacterial cell envelope, which is commonly used to probe the activity of bacterial efflux pump^18^. The property of dye that it emits weak fluorescence outside cells and becomes strongly fluorescent once inside the cells, owing to its binding with DNA^19^, was exploited as a mean to study the integrity of cell envelope. *M*. *tuberculosis* cells expressing full-length YidC(Mtb) and empty-plasmid containing control were incubated with 50 ng/ml ATc for 4 days followed by incubation with 6 μM of EtBr for different time points and estimation of fluorescence, as described in the materials and methods. While the control strain exhibits a minimal uptake of EtBr, the YidC-overexpressing cells accumulate ~6-fold more EtBr compared to control at all time points of 15-, 30- and 60 minutes, thus confirming a compromised integrity of mycobacterial cell envelope (Fig. 6A). Remarkably, YidC(Mtb) exhibits a similar damaging effect on cell envelope of *M*. *smegmatis* while its own protein, YidC(Msm) does not affect the cellular permeability barrier to EtBr (Fig. 6B).

To further gain an insight into the effect of YidC(Mtb) on integrity of mycobacterial cell envelope, we next analysed cell morphology of *M*. *tuberculosis* cells by electron microscopy. Broth cultures of the empty plasmid containing control, YidC(Msm)- and YidC(Mtb)*-*overexpressing *M*. *tuberculosis* strains were incubated with 50 ng/ml ATc for 4 days, fixed with paraformaldehyde and subjected to morphological analysis by transmission electron microscopy. We observed that cells expressing YidC(Mtb) exhibit a detectable difference in cell wall structure compared to control or those expressing YidC(Msm) (Fig. 6C). As illustrated in Fig. 6D, overexpression of YidC(Mtb) results in ruptured cell envelope, whereas those harbouring pTetR (Control) or overexpressing YidC(Msm) are intact exhibiting distinct bilayer around the cell.

## Discussion

Although pathogenic mycobacteria survive in the host for decades, only ~10% of immunocompetent individuals essentially develop the disease upon exposure to the pathogen^1^, which indicates that in majority of individuals with intact immunity, host cells are well-equipped to thwart colonization and intracellular proliferation of pathogen. There is continuous cross-talk between host cells and the tubercle bacilli in the infected individuals which eventually decide fate of the disease. Once inside the host, *M*. *tuberculosis* interacts with different cell types such as alveolar macrophages, dendritic cells, lung epithelial cells etc. that together mount a strong anti-mycobacterial response to contain the dissemination of pathogen. However, in order to counter the host assaults bacteria rapidly adapt themselves and modulate the expression of key virulence factors for their survival^9^.

Proteins embedded in the cell envelope play an important role in setting up the stage for host-pathogen interaction, and exhibit differential expression upon infection^10,11,20^. YidC is a cell envelope localized translocase which regulates translocation of key respiratory proteins to cell membrane in *M*. *tuberculosis*, and is indispensable for mycobacterial proliferation *in vitro* as well as in macrophages^14,17^, suggesting its constitutive expression. In an attempt to further characterize the expression profile of *yidC* in *Mycobacterium*, we surprisingly made an observation revealing its transcription as an operon with upstream genes that are primarily involved in ribosome biosynthesis (Fig. 1). Remarkably, the YidC protein shows a moderate induction despite significant downregulation of its transcripts under membrane stress conditions (Fig. 2). In contrast, overexpression of *yidC* mRNA *via* an extra copy of ORF in the plasmid results in significant induction of YidC protein (Fig. 3 and Supplementary Fig. 1). Taken together, these findings imply that under the stress conditions YidC protein is independently controlled in *M*. *tuberculosis* at the transcriptional and the translational levels. Importantly, the above results indicate that YidC is a stable protein with a long half-life, which is sufficiently present in the cell during stress to mask the drop in transcript levels and its downstream impact. Further studies are warranted to test if there is difference in ribosomal occupancy rates of *yidC* transcripts during the wild-type and the stress conditions, which may result in uncorrelated mRNA and protein expression. Since, the cell has loosely controlled the mRNA expression for the *yidC* ORF, it might have dictated the resulting protein levels through tight control of its translation such as by regulating the occupancy of ribosomal subunits^21^. Alternatively, the YidC proteins may have different half-lives as the result of varied protein synthesis and degradation during the stress^22^.

Overexpression of membrane proteins is known to elicit a general stress response^23^. Surprisingly, in our study we do not find a marked effect of YidC(Msm), whereas overexpression of the YidC(Mtb) results in altered envelope proteome of *M*. *smegmatis* (Fig. 3), and causes extreme toxicity (Fig. 4). While both the proteins are 75% identical, we notice subtle differences in their topology and arrangement of putative transmembrane (TM) regions across the cell membrane (Supplementary Fig. 2), which may be responsible for their differential effect on mycobacteria. TMHMM database predicts 5 TM segments in YidC(Mtb), whereas in YidC(Msm) only 4 TM regions are projected with a large portion of sequence after the 2^nd^ TM region inside the membrane (Supplementary Fig. 2B). Likewise, the extreme C-terminus region is proposed outside the membrane in YidC(Mtb), and inside in YidC(Msm) (Supplementary Fig. 2B). Currently, it is not clear how such variabilities would affect the conformation and the translocase activities of the two orthologues resulting in differential consequences of these on mycobacteria. Moreover, our findings also indicate that not all the membrane proteins exert lethal effect upon overexpression.

To further gain an insight into the effect of *yidC(Mtb)* on bacterial growth, we next determined if its induction affects protein synthesis. Our preliminary observations on uptake of puromycin suggested that induction of YidC does not affect protein synthesis. Instead, it was observed that YidC induction essentially results in stimulation of many genes that encode membrane proteins including a subset of genes induced by detergent^16^ (Fig. 5). Importantly, overexpression of YidC(Mtb), and not YidC(Msm), causes a significant overexpression of *35kd_ag*, an orthologue of phage shock protein encoding gene in *M*. *tuberculosis*^24^, which is required for maintaining the integrity of inner membrane under stress in enterobacteria, and its expression is sharply induced in the presence of cyanide m-chlorophenylhydrazone (CCCP), an ionophore which disrupts membrane potential^14,25^. Based on these results, we conclude that sustained incorporation of an excess of YidC(Mtb) into the cell envelope by its overexpression elicits membrane stress response possibly by interfering with the integration and later expression levels of other membrane proteins, leading to adverse effects on growth of bacteria. Altered envelope profile and increased permeability to EtBr due to overexpression of full-length protein further confirms the existence of membrane stress in YidC(Mtb)-induced cells (Fig. 6A,B). The adverse effects of YidC(Mtb) induction on mycobacteria was also corroborated by carefully examining the architecture of cell envelope of control and YidC-overexpression strains by electron microscopy, which indicated significant damage of lipid bilayer surrounding the mycobacterial cells due to YidC(Mtb) overexpression (Fig. 6C,D). These findings are in line with a recent observation with *E*. *coli* protein which reveals membrane thinning around YidC^26^, and further establish that induction of YidC under select stress conditions could be a defensive strategy by the host causing mycobacterial cell damage due to extreme weakening of cell membrane eventually resulting in death of the pathogen. Importantly, none of these phenotypes were observed with YidC(Msm) induction, thus reiterating the specific effect of YidC(Mtb) on mycobacteria (Figs 5 and 6).

Uniquely composed cell envelope of *M*. *tuberculosis* is critical for bacterial virulence and offers targets for many frontline anti-TB drugs such as isoniazid and ethambutol. Previously it was established that YidC(Mtb) regulates the membrane translocation of respiratory chain complexes in *M*. *tuberculosis*. In this study we further show how YidC(Mtb) levels are differentially modulated at the transcriptional and the translational levels upon stress, and affect mycobacterial growth and metabolism upon overexpression.

## Materials and Methods

### Bacterial strains and culturing

The study was performed with *M*. *tuberculosis* H37Ra, an avirulent counterpart of *M*. *tuberculosis* H37Rv, which was kindly provided by Dr. William Bishai at the Johns Hopkins University, USA. *Mycobacterium smegmatis* MC^2^155 was kindly provided by Dr. William R. Jacobs, Jr., Albert Einstein College of Medicine, USA. *M*. *tuberculosis* were cultured in 7H9 broth medium (DIFCO) containing 0.2% glycerol, 0.05% tyloxapol and 1X oleic acid-albumin-dextrose-saline (OADS) supplement, whereas *M*. *smegmatis* was grown in the same broth lacking OADS. Wherever required, we used 50 μg/ml hygromycin for culturing recombinant mycobacterial strains. All the plasmids and primers used in this study are listed in Table 1.

### Preparation of plasmid clones

Primer pairs Pr3 and Pr5, bearing *Nde I* and *Hind III* sites respectively, were used to PCR amplify *MRA_3960* from *M*. *tuberculosis* genomic DNA, whereas MS6942_F & MS6942_R were used to amplify *MSMEG_6942* using genomic DNA of *M*. *smegmatis*. Both the PCR products of ~1.1 kb were gel purified, extracted and ligated into pGEM-T^®^ Easy plasmid (Promega) by TA cloning followed by confirmation of correct clone by sequencing.

For ATc-inducible expression of *yidC(Msm)* and *yidC(Mtb)*, the *Nde I* and *Hind III* digested *MSMEG_6942* and *MRA_3960* fragments, obtained from respective pGEM clones, were inserted in pTetR^17^ at the same sites downstream to tetracycline-inducible promoter, *P*_*myc1TetO*_; the recombinant plasmids were annotated as pTetR-*yidC(Msm)* and pTetR-*yidC(Mtb)*, respectively (Table 1).

### RNA extraction and microarray analysis

Total RNA was extracted using TRizol (Invitrogen Life Technologies) as described previously^14^ from control and *yidC*-overexpressing cells of *M*. *tuberculosis* after 4 days of treatment with 50 ng/ml ATc. Preparation of cDNA and labelling was performed using 4 μg RNA from both the strains with the help of SuperScript® Direct cDNA Labeling System (Thermo Fisher). For each hybridization, the complementary DNA (cDNA) probes were labelled with Alexa fluor 555 or Alexa fluor 647 (Life Technologies) and used in pairs. Microarray slide specific to *M*. *tuberculosis* was commercially obtained from Microarray Inc., USA which contains oligonucleotides specific to 3924 genes representing complete genome of *M*. *tuberculosis* in triplicates. Scanning of slides and data analyses were performed as described previously^14^. Briefly, mean fluorescence intensities of each of the 3924 spots from three different hybridizations per array were analysed after normalization with total fluorescence intensities. Fold-change in expression levels was subsequently determined using the normalized fluorescence intensities of green (control) and red (*yidC*-overexpression) channels. Genes that exhibit >2-fold difference in expression between the two strains in three biological replicates with *P* values of 0.05 or less, as determined by paired Student’s *t* test and false discovery rate (FDR) of ≤5% were considered for further analysis. FDR for differentially expressed genes was calculated by Benjamini-Hochberg procedure.

### Reverse transcription and amplification of cDNA

First strand cDNA was synthesized with 500 ng total RNA after DNase I-treatment using random hexamer primers and Superscript III RT (Invitrogen Life Technologies), as per the manufacturer’s recommendations. For operon analysis, 50 ng cDNA was subject to PCR amplification using Taq polymerase (Promega) with the following conditions for each of the 35 cycles: denaturation at 95 °C for 10 seconds, primer annealing at 55 °C for 20 seconds and amplification at 72 °C for 1 minute.

### Quantitative reverse transcription (RT) real-time PCR (qRT-PCR)

First strand cDNA was synthesized as described above. PCR was performed with 50 ng cDNAs and SYBR Green PCR Master Mix (Applied Biosystems) with the help of gene-specific primer pairs (Table 1) and real-time quantification was carried out using the ABI 7500 Fast Real-Time PCR System (Applied Biosystems) as instructed by the manufacturer. Expression level of different genes in *yidC*-overexpression strain was estimated relative to their expression in control after normalizing with the change in expression level of a housekeeping gene *sigA*, as reported previously^14^.

### Ethidium bromide uptake

Uptake of EtBr was measured according to the previous protocol with some modifications^18^. *M*. *tuberculosis* cells harbouring either of pTetR (Control) or pTetR-*yidC(Mtb)* [YidC(Mtb)] were grown to an OD_600_ of 0.2 and cultured in the presence of 50 ng/ml ATc for four days. Similarly, for estimation of EtBr uptake in *M*. *smegmatis*, bacteria containing either of pTetR-*yidC(Mtb)* [YidC(Mtb)] or pTetR-*yidC(Msm)* [YidC(Msm)] were cultured in the presence of 50 ng/ml ATc for 24 hours. Subsequently, cells were pelleted, washed once with PBS containing 0.05% tyloxapol and OD_600_ of each culture suspension in PBS-0.05% tyloxapol was adjusted to 0.4, followed by incubation with 6 μM of EtBr for different time points. Finally, cells were centrifuged at 8000 rpm for 5 minutes, washed once and re-suspended in equal volume of PBS-0.05% tyloxapol and transferred to 96-well plate for fluorescent measurement in a Synergy HT plate reader (Bio-Tek) according to the manufacturer’s instructions. Fluorescence was measured at the excitation wavelength of 530 nm and an emission wavelength of 590 nm. Culture suspensions without EtBr incubation were used as control for normalization of auto-fluorescence.

### Preparation of samples for electron microscopy

Cultures of both the control and YidC-overexpression strains of *M*. *tuberculosis* were incubated with 50 ng/ml ATc for four days, followed by centrifugation and washing of bacterial culture pellets with 0.1 M phosphate buffer. Subsequently, cells were fixed in 2.5% glutaraldehyde and 2% paraformaldehyde in 0.1 M phosphate buffer for 4 hours at 4 °C, washed twice with 0.1 M phosphate buffer and suspended in the same buffer for preparation of blocks. Block preparation, sectioning and sample viewing were performed at the sophisticated analytical instrumentation facility in the All India Institute of Medical Sciences, New Delhi (http://www.saifaiims.com/reg-info.php).

### Immunoblotting

Whole cell extracts of mycobacteria were prepared by bead-beating lysis in PBS and immunoblotting was performed with 20 μg of bacterial WCEs (unless specifically mentioned) using anti-YidC antibodies, as reported earlier^14,17^.

### Accession codes

Gene expression data have been deposited in GEO database under the accession code GSE110355.

## Electronic supplementary material

Supplementary Information

## References

[1] WHO. Bending The Curve-ending TB: Annual TB Report 2017. (World Health Organization, Geneva, 2017).

[2] Weiss G, Schaible UE (2015). Macrophage defense mechanisms against intracellular bacteria. Immunol Rev.

[3] Russell DG (2001). Mycobacterium tuberculosis: here today, and here tomorrow. Nat Rev Mol Cell Biol.

[4] Creuwels LA, van Golde LM, Haagsman HP (1997). The pulmonary surfactant system: biochemical and clinical aspects. Lung.

[5] Gaynor CD, McCormack FX, Voelker DR, McGowan SE, Schlesinger LS (1995). Pulmonary surfactant protein A mediates enhanced phagocytosis of Mycobacterium tuberculosis by a direct interaction with human macrophages. J Immunol.

[6] Ryndak MB, Singh KK, Peng Z, Laal S (2015). Transcriptional Profiling of Mycobacterium tuberculosis Replicating in the Human Type II Alveolar Epithelial Cell Line, A549. Genom Data.

[7] Ryndak MB, Singh KK, Peng Z, Laal S (2015). Transcriptional profile of Mycobacterium tuberculosis replicating in type II alveolar epithelial cells. PLoS One.

[8] Schwab U (2009). Transcriptional responses of Mycobacterium tuberculosis to lung surfactant. Microb Pathog.

[9] Honer zu Bentrup K, Russell DG (2001). Mycobacterial persistence: adaptation to a changing environment. Trends Microbiol.

[10] Feltcher ME, Sullivan JT, Braunstein M (2010). Protein export systems of Mycobacterium tuberculosis: novel targets for drug development?. Future Microbiol.

[11] Simeone R, Bottai D, Frigui W, Majlessi L, Brosch R (2015). ESX/type VII secretion systems of mycobacteria: Insights into evolution, pathogenicity and protection. Tuberculosis (Edinb).

[12] Abdallah AM (2007). Type VII secretion–mycobacteria show the wa. y. Nat Rev Microbiol.

[13] Feltcher ME (2015). Label-free Quantitative Proteomics Reveals a Role for the Mycobacterium tuberculosis SecA2 Pathway in Exporting Solute Binding Proteins and Mce Transporters to the Cell Wall. Mol Cell Proteomics.

[14] Thakur P (2016). The preprotein translocase YidC controls respiratory metabolism in Mycobacterium tuberculosis. Sci Rep.

[15] Geiman DE, Raghunand TR, Agarwal N, Bishai WR (2006). Differential gene expression in response to exposure to antimycobacterial agents and other stress conditions among seven Mycobacterium tuberculosis whiB-like genes. Antimicrob Agents Chemother.

[16] Manganelli R, Voskuil MI, Schoolnik GK, Smith I (2001). The Mycobacterium tuberculosis ECF sigma factor sigmaE: role in global gene expression and survival in macrophages. Mol Microbiol.

[17] Choudhary E, Thakur P, Pareek M, Agarwal N (2015). Gene silencing by CRISPR interference in mycobacteria. Nat Commun.

[18] Rodrigues L, Ramos J, Couto I, Amaral L, Viveiros M (2011). Ethidium bromide transport across Mycobacterium smegmatis cell-wall: correlation with antibiotic resistance. BMC Microbiol.

[19] Sharples D, Brown JR (1976). Correlation of the base specificity of DNA–intercalating ligands with their physico-chemical properties. Febs lett.

[20] Simeone R, Bottai D, Brosch R (2009). ESX/type VII secretion systems and their role in host-pathogen interaction. Curr Opin Microbiol.

[21] Arava Y (2003). Genome-wide analysis of mRNA translation profiles in Saccharomyces cerevisiae. Proc Natl Acad Sci USA.

[22] Greenbaum D, Colangelo C, Williams K, Gerstein M (2003). Comparing protein abundance and mRNA expression levels on a genomic scale. Genome Biol.

[23] Wagner S (2007). Consequences of membrane protein overexpression in Escherichia coli. Mol Cell Proteomics.

[24] Datta P (2015). The Psp system of Mycobacterium tuberculosis integrates envelope stress-sensing and envelope-preserving functions. Mol Microbiol.

[25] McDonald C, Jovanovic G, Ces O, Buck M (2015). Membrane Stored Curvature Elastic Stress Modulates Recruitment of Maintenance Proteins PspA and Vipp1. MBio.

[26] Chen Y (2017). YidC Insertase of Escherichia coli: Water Accessibility and Membrane Shaping. Structure.

