# Supplementary material for: Regulation and overexpression studies of YidC in Mycobacterium tuberculosis
Source: Sci Rep. 2018 Nov 20;8:17114. doi: 10.1038/s41598-018-35475-4 (PMC6244158; doi:10.1038/s41598-018-35475-4)
Supplement: Supplementary file 1 — Supplementary Information [file 41598_2018_35475_MOESM1_ESM.pdf]

<u>SUPPLEMENTARY INFORMATION</u>

Regulation and overexpression studies of YidC in *Mycobacterium tuberculosis*

Preeti Thakur, Eira Choudhary, Madhu Pareek and Nisheeth Agarwal*

Key words: Mycobacterium tuberculosis; YidC; cell surface stress

Running Title: Effect of YidC overexpression on M. tuberculosis

***To whom the correspondence should be addressed**

Affiliation:
Translational Health Science and Technology Institute,
NCR Biotech Science Cluster,
3rd Milestone, Faridabad–Gurgaon Expressway,
Faridabad- 121001 (Haryana)
Phone No. 91-129-2876304

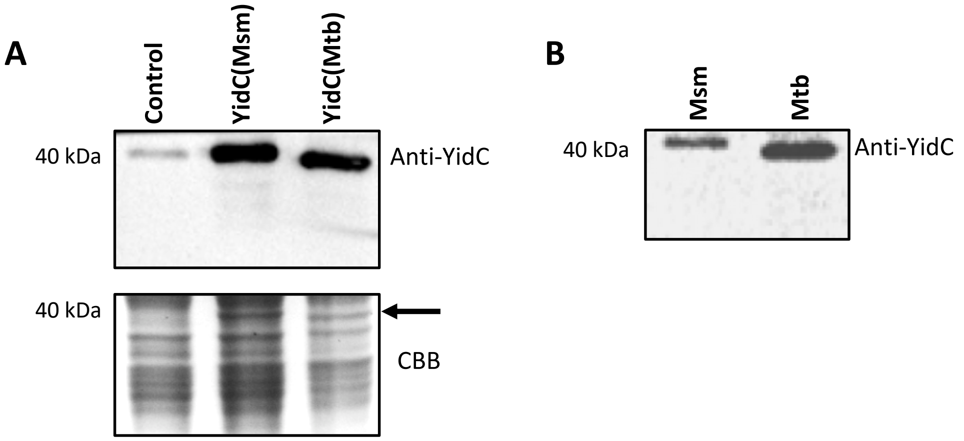

Supplementary Fig. 1. Comparative analysis of YidC(Mtb) and YidC(Msm) expression. YidC expression was analyzed by anti-YidC immunoblotting using the whole cell extracts of YidC-overexpression *(A)* and wild-type *(B)* strains. Control, YidC(Msm) and YidC(Mtb) in *(A)* denote *M. smegmatis* harboring ptetR, pTetR-*yidC(Msm)* and pTetR-*yidC(Mtb)* respectively, whereas Msm and Mtb in *(B)* represent wild-type *M. smegmatis* and *M. tuberculosis*. Arrow in CBB-stained gel marks the position of respective YidC variant *(A)*. Immunoblotting was performed using 5µg lysates in *(A)* and 20µg lysates in *(B),* respectively.

A

```
YidC(Mtb)    4   LFDFFSLDFIYYPVSWIMWVWYRLFAFVLGPSNFFAWALSVMFLVFTLRALLYKPFVRQI  63
                 +F+FFSLD IYYPVS IMWVWY+ F+F+LGP+NFFAWALSVMFLVFTLRA+LYKPFV+QI
YidC(Msm)    1   MFNFFSLDIIYYPVSAIMWVWYKAFSFLLGPTNFFAWALSVMFLVFTLRAILYKPFVKQI  60

YidC(Mtb)   64.  RTTRQMQELQPQIKALQKKYGKDRQRMALEMQKLQREHGFNPILGCLPMLAQIPVFLGLY  123
                 RTTRQMQELQPQIKALQKKYGKDRQRMALEMQKLQ+EHGFNPILGCLPMLAQ+PVFLGLY
YidC(Msm)   61   RTTRQMQELQPQIKALQKKYGKDRQRMALEMQKLQKEHGFNPILGCLPMLAQVPVFLGLY  120

YidC(Mtb)  124   HVLRSFNRTTGGFGQPHLSVIENRLTGNYVFSPVDVGHFLDANLFGAPIGAYMTQRSGLD  183
                 HVL SFNRT  G G+  LSV ENR  GNYVFS  DV HFLDANLFGAP+GA M Q+ GL+
YidC(Msm)  121   HVLMSFNRTQTGIGRLGLSVEENRSLGNYVFSATDVQHFLDANLFGAPLGATMIQQHGLE  180

YidC(Mtb)  184   AFVDFSRPALIAVGVPVMILAGIATYFNSRASIARQSAEAAANPQTAMMNKLALYVFPLG  243
                 AF +F+R A+I VGVP+MILAGIAT+FNSRAS+ARQS EAAANPQTAMMNKLALYVFPLG
YidC(Msm)  181   AFTEFNRLAVIIVGVPIMILAGIATHFNSRASVARQSVEAAANPQTAMMNKLALYVFPLG  240

YidC(Mtb)  244   VVVGGPFLPLAIILYWFSNNIWTFGQQHYVFGMIEKEEEAKKQEAVRRRAANAPAPGAKP  303
                 VVVGGPFLPLA+I+YW +NNIWT+GQQHYVFG IEKEEEAKK E + RRAANAPAPGAKP
YidC(Msm)  241   VVVGGPFLPLAVIMYWLANNIWTYGQQHYVFGKIEKEEEAKKAEMLERRAANAPAPGAKP  300

YidC(Mtb)  304   KRSPKTAPATNAAAPTEAGD-TDDGAESDASTERPADT-SNPARRNSG--PSARTPRPGV  359
                  R  K  P   A A  EAG  T+   ES  ++E   D+ +  A   SG  P+ RTP+PG
YidC(Msm)  301   NRMKKAQPGGQAGAEGEAGSATELTGESSGTSEPTTDSGATGASGTSGTTPTNRTPKPGA  360

YidC(Mtb)  360   RPKKRKR  366
                 RPKKRKR
YidC(Msm)  361   RPKKRKR  367
```

B

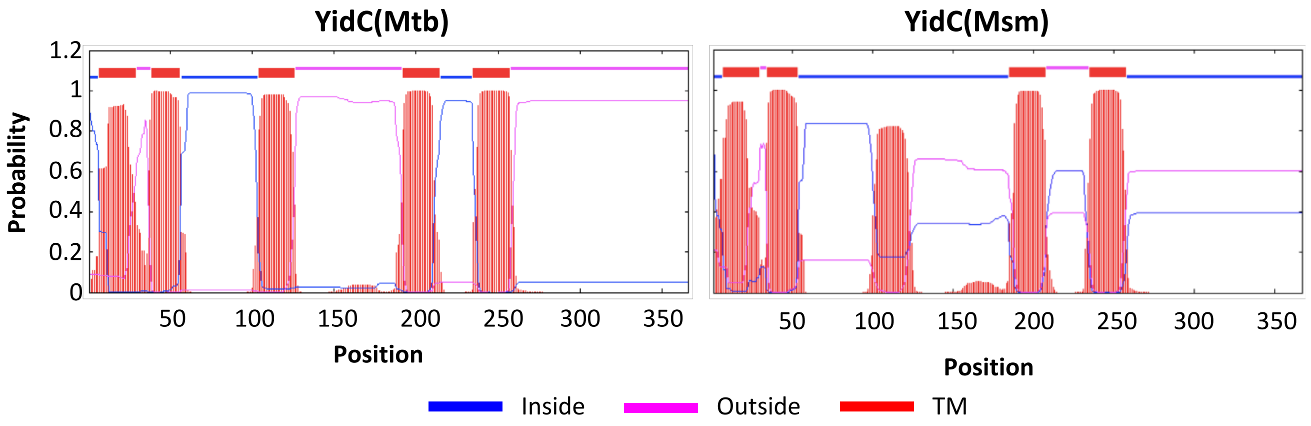

Supplementary Fig. 2. Comparative analysis of YidC(Mtb) and YidC(Msm). Amino acid sequences of both the proteins were compared by BLASTp *(A)* and by TMHMM database *(B)*, using the default parameters.
